# QuickStats

**Published:** 2014-06-13

**Authors:** FROM THE NATIONAL CENTER FOR HEALTH STATISTICS

**Figure:**
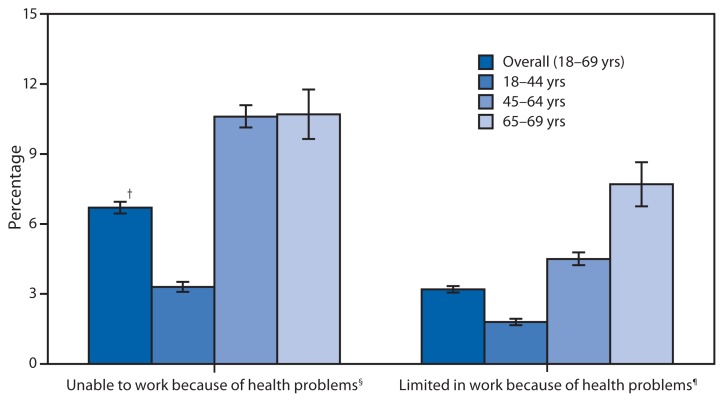
Percentage of Adults Aged 18–69 Years With a Limitation in Their Ability to Work Because of Health Problems, by Age Group — National Health Interview Survey,* United States, 2012 * Estimates are based on household interviews of a sample of the civilian, noninstitutionalized U.S. population. Persons with unknown work limitation status were excluded from the denominators. ^†^ 95% confidence interval. ^§^ Based on responses to the question, “Does a physical, mental, or emotional problem now keep [family members aged ≥18 years] from working at a job or business?” Respondents were asked to answer regarding themselves and other family members living in the same household. ^¶^ For persons able to work, based on responses to the question, “Are [family members aged ≥18 years] limited in the kind or amount of work they can do because of a physical, mental, or emotional problem?” Respondents were asked to answer regarding themselves and other family members living in the same household.

In 2012, approximately 7% of adults aged 18–69 years were unable to work, and approximately 3% were limited in their ability to work because of health problems. Adults aged 45–64 years and 65–69 years were about three times more likely than adults aged 18–44 years to be unable to work because of health problems. The percentage of adults limited in their ability to work because of health problems also increased with age.

**Source:** Adams PF, Kirzinger WK, Martinez ME. Summary health statistics for the U.S. population: National Health Interview Survey; 2012. Vital Health Stat 2013;10(259).

**Reported by:** Patricia F. Adams, pfa1@cdc.gov, 301-458-4063; Whitney K. Kirzinger, MPH; Michael E. Martinez, MPH, MHSA.

